# Performance of an artificial intelligence-guided quantitative coronary computed tomography algorithm for predicting myocardial ischemia in real-world practice

**DOI:** 10.1016/j.ijcha.2024.101433

**Published:** 2024-05-31

**Authors:** Ronald P. Karlsberg, Nick S. Nurmohamed, Carlos G. Quesada, Bruce A. Samuels, Suhail Dohad, Lauren R. Anderson, Tami Crabtree, James K. Min, Andrew D. Choi, James P. Earls

**Affiliations:** a Cardiovascular Research Foundation of Southern California – Cedars-Sinai Smidt Heart Institute, Los Angeles, CA, USA; b The George Washington University School of Medicine, Washington, DC, USA; c Department of Cardiology, Amsterdam UMC, Vrije Universiteit Amsterdam, Amsterdam, the Netherlands; d Department of Vascular Medicine, Amsterdam UMC, University of Amsterdam, Amsterdam, the Netherlands; e Cleerly Inc., Denver, CO, USA

Traditionally, noninvasive stress testing with nuclear perfusion imaging modalities as single-photon emission computed tomography (SPECT) and position emission tomography (PET) has been the gold standard for detection of myocardial ischemia [1]. Up to 50 % of patients suspected of CAD has myocardial ischemia in at least one vascular territory according to [^15^O]H_2_O PET [2]. However, these approaches are associated with a high cost as well as radiation burden, in contrast to coronary CT angiography (CCTA)[3], [4]. Therefore, recent efforts have investigated the use of anatomical characteristics from CCTA analyzed by artificial intelligence (Atherosclerosis Imaging-Quantitative Computed Tomopgraphy [AI-QCT], Cleerly Inc, Denver, CO)[5], [6], [7], [8] to analyze the probability of vessel-specific coronary ischemia. In two controlled research settings, this novel algorithm (AI-QCT_ISCHEMIA_) showed high accuracy for predicting reduced fractional flow reserve (FFR), at least similar to FFR_CT_, and has shown important value for major adverse cardiovascular events prognostication [9], [10]. The present analysis aimed to serve as a novel investigation of the diagnostic accuracy of the recently developed algorithm for prediction of vessel-based ischemia in a real-world setting.

In this single center study, patients suspected of coronary artery disease (CAD) who underwent both CCTA as well as invasive coronary angiography were included. CCTA exams were analyzed using the AI-QCT algorithm including AI-QCT_ISCHEMIA._ As described previously, the AI-QCT_ISCHEMIA_ algorithm utilizes 37 parameters of stenosis, plaque characterization, plaque diffuseness and vascular morphology derived from AI-QCT in a random forest model to predict the presence of myocardial ischemia as defined by a reduced FFR ≤ 0.8.^9^ AI-QCT_ISCHEMIA_ was previously developed, internally and externally validated in the multicenter Computed Tomographic Evaluation of Atherosclerotic Determinants of Myocardial Ischemia (CREDENCE) and Prospective Comparison of Cardiac PET/CT, SPECT/CT Perfusion Imaging and CT Coronary Angiography With Invasive Coronary Angiography (PACIFIC-1) trials. In the present study, the performance of AI-QCT_ISCHEMIA_ was compared to fractional flow reserve derived from CCTA (FFR_CT_ Heartflow Inc, Redwood City, CA for the prediction of a reduced invasive FFR (FFR ≤ 0.8) or instantaneous wave-free ratio (iFR ≤ 0.89) as reference standard for invasive vessel-based ischemia. The analysis was restricted to vessels with an AI-QCT_ISCHEMIA_, FFR_CT_ and invasive measurement available in the main epicardial coronary vessels (left main, anterior descending coronary artery, right coronary artery, and circumflex coronary artery). Predictive performances were assessed using measures of accuracy (sensitivity, specificity and overall accuracy) an area under the receiver operating characteristic curve (AUC) analysis, both on a per-vessel and a per-patient basis. AUCs were compared using a DeLong test. All statistical analyses were performed using SAS software version 9.4 (SAS Institute Inc., Cary, NC, USA).

A total of 35 patients and 42 vessels were included in the analysis. Mean age was 69.4 ± 10.0 years, 9 (26 %) patients were female, 22 (63 %) patients had hypertension, 4 (11 %) patients had diabetes, 17 (49 %) patients had a history of smoking and 29 (83 %) patients had dyslipidemia. AI-QCT_ISCHEMIA_ achieved a sensitivity of 82 %, a specificity 76 % and an accuracy of 79 % for predicting vessel-based ischemia. For FFR_CT_, these measures were 94 %, 64 % and 44 %, respectively (Table 1). The AUCs were comparable between the two approaches: AI-QCT_ISCHEMIA_ achieved an AUC of 0.87 and FFR_CT_ achieved an AUC of 0.85 (p = 0.825). Specificity was higher for AI-QCT_ISCHEMIA_ when compared to FFR_CT_ (76 % vs 44 %; p = 0.031). On a per-patient basis, AI-QCT_ISCHEMIA_ achieved a sensitivity of 88 %, a specificity 79 % and an accuracy of 83 % for predicting ischemia. For FFR_CT_, these measures were 88 %, 47 % and 79 %, respectively. Again, AI-QCT_ISCHEMIA_ and FFR_CT_ achieved a similar AUC (0.81 vs. 0.89; p = 0.406). A case example showing the concordance between AI-QCT_ISCHEMIA_, FFR and FFR_CT_ is shown in Fig. 1.

**Table 1:** Performance of AI-QCT_ISCHEMIA_ versus FFR_CT_ for predicting of coronary ischemia.

Endpoint	AI-QCT_ISCHEMIA_	FFR_CT_	p-value
*Per-vessel*			
Sensitivity	82 % (14/17)	94 % (16/17)	0.308
Specificity	76 % (19/25)	44 % (11/25)	0.031
Accuracy	79 % (33/42)	64 % (27/42)	0.133
AUC	0.87	0.85	0.825

*Per-patient*			
Sensitivity	88 % (14/16)	88 % (14/16)	1.000
Specificity	79 % (15/19)	47 % (9/19)	0.083
Accuracy	83 % (29/35)	66 % (23/35)	0.134
AUC	0.81	0.89	0.406

AUC, area under the curve, AI-QCT, atherosclerosis imaging-quantitative computed tomography; FFR_CT_, fractional flow reserve from CT.

**Fig. 1:**
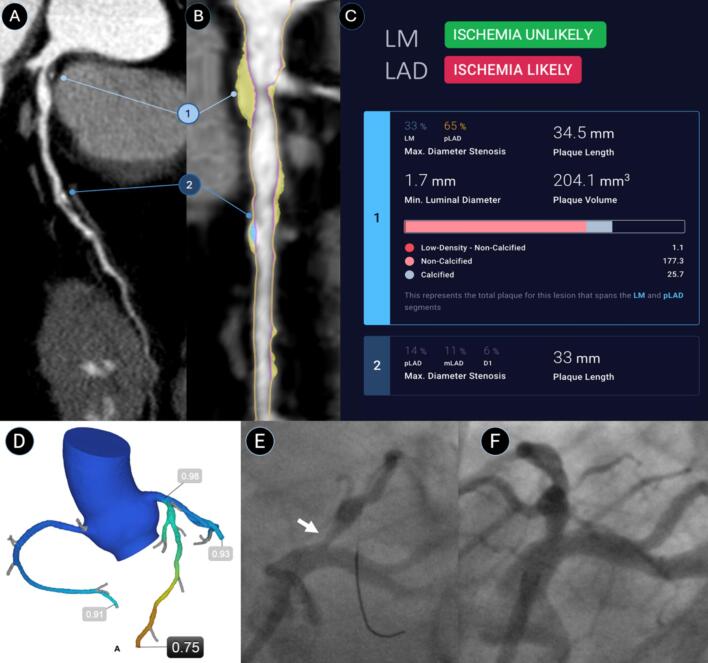
**AI-QCT_ISCHEMIA_ case example.** 50-year old male with new-onset stable chest pain. CCTA curved reformat depicts plaque in the LM and LAD (A). AI-QCT depicts predominantly non-calcified plaque (yellow overlay) in the LM/pLAD and mixed plaque (blue overlay) in the mid vessel (B). AI-QCT_ISCHEMIA_ depicts 2 plaques, one extending from the LM (33% stenosis) to the proximal LAD (65% stenosis), the second plaque has a maximal stenosis of 14% (C). AI-QCT_ISCHEMIA_ determined ischemia likely in the LAD, but not in the LM. This was concordant with a reduced FFR_CT_ of 0.75 (D). It was also concordant with invasive angiography where both a 70% stenosis as well as a reduced invasive FFR of 0.67 were found in the pLAD (E). The patient underwent successful placement of a drug-eluting stent with TIMI-3 flow result (F). AI-QCT, atherosclerosis imaging-quantitative coronary computed tomography; CCTA, coronary CT angiography; FFR, fractional flow reserve; FFR_CT_, fractional flow reserve from CT; LM, left main; LAD, left anterior descending; TIMI, Thrombolysis in Myocardial Infarction. (For interpretation of the references to colour in this figure legend, the reader is referred to the web version of this article.)

In this real-world analysis, AI-QCT_ISCHEMIA_ showed high diagnostic accuracy and high specificity for vessel-specific ischemia in this population. These data demonstrate the feasibility of artificial intelligence-guided CCTA for predicting presence of functional myocardial ischemia based on anatomical characteristics, and thus its potential to expand its use as a 3-in-1 approach including assessment of ischemia beyond atherosclerosis and stenosis alone. The current single center study was limited by a small sample size, and lack of a standardized protocol for invasive ischemia assessment (i.e. both FFR and iFR were used), due to the real-world setting. Results may vary in study populations without symptoms, less disease, and a lower incidence of patients with parameters of ischemia. Future larger, multicenter studies are required to further investigate the performance of this novel algorithm.

## CRediT authorship contribution statement

**Ronald P. Karlsberg:** Writing – review & editing, Writing – original draft, Resources, Project administration, Methodology, Data curation, Conceptualization. **Nick S. Nurmohamed:** Writing – review & editing, Writing – original draft. **Carlos G. Quesada:** Writing – review & editing, Data curation. **Bruce A. Samuels:** Writing – review & editing, Data curation. **Suhail Dohad:** Writing – review & editing, Data curation. **Lauren R. Anderson:** Writing – review & editing, Data curation. **Tami Crabtree:** Writing – review & editing, Formal analysis. **James K. Min:** Writing – review & editing. **Andrew D. Choi:** Writing – review & editing, Methodology. **James P. Earls:** Writing – review & editing, Visualization.

## Declaration of competing interest

NSN reports grants from the Dutch Heart Foundation (Dekker 03–007-2023–0068), European Atherosclerosis Society (2023), research funding/speaker fees from Cleerly, Daiichi Sankyo and Novartis, and is co-founder of Lipid Tools. ADC reports grant support from GW Heart and Vascular Institute, equity in Cleerly, Inc and consulting with Siemens Healthineers. TC, JKM and JPE are employees of Cleerly Inc. The other authors report no relevant disclosures.

## References

[1] Gulati M., Levy P.D., Mukherjee D., Amsterdam E., Bhatt D.L., Birtcher K.K., Blankstein R., Boyd J., Bullock-Palmer R.P., Conejo T., Diercks D.B., Gentile F., Greenwood J.P., Hess E.P., Hollenberg S.M., Jaber W.A., Jneid H., Joglar J.A., Morrow D.A., O’Connor R.E., Ross M.A., Shaw L.J. (2021). 2021 AHA/ACC/ASE/CHEST/SAEM/SCCT/SCMR Guideline for the evaluation and diagnosis of chest pain: a report of the American college of cardiology/American heart association joint committee on clinical practice guidelines. *J. Am. Coll. Cardiol*.

[2] Danad I., Raijmakers P.G., Driessen R.S., Leipsic J., Raju R., Naoum C., Knuuti J., Mäki M., Underwood R.S., Min J.K., Elmore K., Stuijfzand W.J., Van R.N., Tulevski I.I., Somsen A.G., Huisman M.C., Van L.AA., Heymans M.W., Van De V.PM., Van K.C., Lammertsma A.A., Van R.AC., Knaapen P. (2017). Comparison of coronary CT angiography, SPECT, PET, and hybrid imaging for diagnosis of ischemic heart disease determined by fractional flow reserve. JAMA Cardiol..

[3] Einstein A.J., Berman D.S., Min J.K., Hendel R.C., Gerber T.C., Carr J.J., Cerqueira M.D., Cullom S.J., Dekemp R., Dickert N.W., Dorbala S., Fazel R., Garcia E.V., Gibbons R.J., Halliburton S.S., Hausleiter J., Heller G.V., Jerome S., Lesser J.R., Raff G.L., Tilkemeier P., Williams K.A., Shaw L.J. (2014). Patient-centered imaging: Shared decision making for cardiac imaging procedures with exposure to ionizing radiation. J. Am. Coll. Cardiol..

[4] Nurmohamed N.S., van Rosendael A.R., Danad I., Ngo-Metzger Q., Taub P.R., Ray K.K., Figtree G., Bonaca M.P., Hsia J., Rodriguez F., Sandhu A.T., Nieman K., Earls J.P., Hoffmann U., Bax J.J., Min J.K., Maron D.J., Bhatt D.L. (2024). Atherosclerosis evaluation and cardiovascular risk estimation using coronary computed tomography angiography. *Eur. Heart J.*.

[5] Nurmohamed N.S., Cole J.H., Budoff M., Karlsberg R.P., Gupta H., Sullenberger L.E., Quesada C.G., Rahban H., Woods K.M., Uzzilia J.R., Purga S.L., Aquino M., Hoffmann U., Min J.K., Earls J.P., Choi A.D. (2024). Impact of atherosclerosis imaging-quantitative computed tomography on diagnostic certainty, downstream testing, coronary revascularization and medical therapy: the CERTAIN study. *Eur. Hear. J. – Cardiovasc. Imaging*.

[6] Nurmohamed N.S., Bom M.J., Jukema R.A., de Groot R.J., Driessen R.S., van Diemen P.A., de Winter R.W., Gaillard E.L., Sprengers R.W., Stroes E.S.G., Min J.K., Earls J.P., Cardoso R., Blankstein R., Danad I., Choi A.D., Knaapen P. (2023). AI-guided quantitative plaque staging predicts long-term cardiovascular outcomes in patients at risk for atherosclerotic CVD. JACC Cardiovasc. Imaging.

[7] Griffin W.F., Choi A.D., Riess J.S., Marques H., Chang H.J., Choi J.H., Doh J.H., Her A.Y., Koo B.K., Nam C.W., Park H.B., Shin S.H., Cole J., Gimelli A., Khan M.A., Lu B., Gao Y., Nabi F., Nakazato R., Schoepf U.J., Driessen R.S., Bom M.J., Thompson R., Jang J.J., Ridner M., Rowan C., Avelar E., Généreux P., Knaapen P., de Waard G.A., Pontone G., Andreini D., Earls J.P. (2023). AI evaluation of stenosis on coronary CTA, comparison with quantitative coronary angiography and fractional flow reserve: a CREDENCE trial substudy. JACC Cardiovasc. Imaging.

[8] Bär S., Maaniitty T., Nabeta T., Bax J.J., Earls J.P., Min J.K., Saraste A., Knuuti J. (2024). Prognostic value of a novel artificial intelligence-based coronary CTA-derived ischemia algorithm among patients with normal or abnormal myocardial perfusion. *J. Cardiovasc. Comput. Tomogr.*.

[9] N.S. Nurmohamed D. Ibrahim R.A. Jukema R. de Winter R.J. de Groot R. Driessen M.J. Bom P.A. van Diemen G. Pontone D. Andreini C. Hyuk-Jae J. KR S.G. SE Hao W, Chung C, Tami C, Melissa A, K. MJ, P. EJ, J. BJ, D. CA, Paul K, R. van RA, Ran H, Hyung-Bok P, Hugo M, J. SW, Hyun CJ, Joon-Hyung D, Ae-Young H, Bon-Kwon K, Chang-Wook N, Sang-Hoon S, Jason C, Alessia G, Akram KM, Bin L, Yang G, Faisal N, H. A-MM, Ryo N, Joseph SU, C. TR, J. JJ, Michael R, Chris R, Erick A, Philippe G, A. de WG, W. SR, Development and Validation of a Quantitative Coronary CT Angiography Model for Diagnosis of Vessel-Specific Coronary Ischemia. *JACC Cardiovasc Imaging* 2024;0. Available at: https://doi.org/10.1016/j.jcmg.2024.01.007. 10.1016/j.jcmg.2024.01.007 38483420

[10] Baer S., Nabeta T., Maaniitty T., Bax J.J., Saraste A., Earls J., Min J.K., Knuuti J. (2023). Prognostic value of a novel artificial intelligence-based coronary computed tomography angiography-derived ischemia algorithm for patients with suspected coronary artery disease. Eur. Heart J..

